# The development of ^177^Lu-DOTA-CC-PSMA following a unified “Click Chemistry” protocol of synthesizing metal nuclide-conjugated radiopharmaceuticals

**DOI:** 10.1186/s41181-024-00287-7

**Published:** 2024-07-31

**Authors:** Xiaobei Zheng, Shuai Xue, Zhongqi Zhao, Shuxin Jin, Shuhua He, Lina Jia, Zheng Li, Christian Vanhove, Filip De Vos, Zijun Kuang, Tiantian Wang, Sara Neyt, Lan Zhang, Xiao Li

**Affiliations:** 1 Shanghai Institute of Applied Physics, Chinese Academy of Sciences, Shanghai, 201800 China; 2 University of Chinese Academy of Sciences, Beijing, 100049 China; 3 Shanghai Vista Pharmaceutical Technology Co. Ltd, Shanghai, 201800 China; 4 Department of Nuclear Medicine, Pudong Hospital, Fudan University, Shanghai, 201399 China; 5 Institute of Biomedical Engineering and Technology, Faculty of Engineering and Architecture, Ghent University, Ghent, 9000 Belgium; 6 Department of Radiopharmacy, Faculty of Pharmacy, Ghent University, Ghent, 9000 Belgium

**Keywords:** Click chemistry, Alkyne, Azido, PSMA, Prostate cancer, ^177^Lu-DOTA-CC-PSMA

## Abstract

**Background:**

Currently, the synthesis pathway of metal nuclide-labeled radiopharmaceuticals is mainly divided into two steps: first, connecting the chelator with the target molecule, and second, labeling the metal nuclide to the chelator. However, the second step of the reaction to label the metal nuclide requires high temperature (90–100 °C), which tends to denature and inactivate the target molecule, leading to loss of biological activities, especially the targeting ability. A feasible solution may be the click chemistry labeling method, which consists of reacting a metal nuclide with a chelating agent to generate an intermediate and then synthesizing a radiopharmaceutical agent via the click chemistry intermediate and the target molecule-alkyne compound. In this study, through the click chemistry of ^177^Lu-DOTA-N_3_ with prostate-specific membrane antigen (PSMA)-alkyne compound, ^177^Lu-labeled PSMA-targeted molecular probe was synthesized and evaluated for its potential to be cleared from the bloodstream and rapidly distributed to tissues and organs, achieving a high target/non-target ratio. ^177^Lu-PSMA-617 was utilized as an analogue for comparison in terms of synthesizing efficiency and PSMA-targeting ability.

**Results:**

A novel ^177^Lu-labeled PSMA radioligand was successfully synthesized through the click chemistry of ^177^Lu-DOTA-N_3_ with PSMA-alkyne compound, and abbreviated as ^177^Lu-DOTA-CC-PSMA, achieving a radiochemical yield of 77.07% ± 0.03% (*n* = 6) and a radiochemical purity of 97.62% ± 1.49% (*n* = 6) when purified by SepPak C18 column. Notably, ^177^Lu-DOTA-CC-PSMA was characterized as a hydrophilic compound that exhibited stability at room temperature and commendable pharmacokinetic properties, such as the superior uptake (19.75 ± 3.02%ID/g at 0.5 h) and retention (9.14 ± 3.16%ID/g at 24 h) within xenografts of 22Rv1 tumor-bearing mice. SPECT/CT imaging indicated that radioactivity in both kidneys and bladder was essentially eliminated after 24 h, while ^177^Lu-DOTA-CC-PSMA was further enriched and retained in PSMA-expressing tumors, resulting in the high target/non-target ratio.

**Conclusion:**

This study demonstrated the potential of click chemistry to unify the synthesis of metal radiopharmaceuticals, and ^177^Lu-DOTA-CC-PSMA was found for rapid clearance and appropriate chemical stability as a PSMA-targeted radioligand.

**Supplementary Information:**

The online version contains supplementary material available at 10.1186/s41181-024-00287-7.

## Background

The main aim of radiopharmaceutical therapy is to form targeted radiopharmaceuticals by linking therapeutic radioisotopes to targeting molecules that precisely identify tumor cells and bind to certain receptors of the tumor cells (Sgouros et al. 2020; Dhoundiyal et al. 2024; Salerno et al. 2023). The radioisotope accumulates and decays at the tumor site, releasing a certain amount of ionizing radiation, which destroys the tumor tissue; meanwhile, the precise positioning allows for targeted therapy with minimal potential impact on surrounding healthy tissues. Prostate-specific membrane antigen (PSMA), a type II transmembrane glycoprotein consisting of 750 amino acids, is commonly overexpressed in almost all prostate cancer cells (Kiess et al. 2015; Haberkorn et al. 2016; Osborne et al. 2013). In contrast, it is expressed at low levels in normal tissues such as kidneys, salivary glands, and small intestine (He et al. 2022); thus, PSMA-targeted radioligand therapy (PRLT) has become a popular therapy for the treatment of prostate cancer (Cimadamore et al. 2018; Wang et al. 2022, 2023).

The synthesis of metal nuclide-based radiopharmaceuticals is generally divided into two steps: first, connecting the chelating agent with the target molecule to form a precursor (e.g., PSMA-617, PSMA-HYNIC, and FAPI-04, which are common in clinic), and then, labeling the radioisotope on the precursor. The target molecules, especially antibodies and some heat-sensitive peptides with complex spatial structures, are prone to denaturation and inactivation at high temperatures or rigor reaction conditions. However, the second step of the reaction usually requires high temperatures or weak acidic conditions, leading to problems such as loss of targeting. A possible way to overcome these problems is to bring forward the labeling reaction of metal radionuclides as a pre-reaction of radio-labeled bioconjugate to bioactive molecules. The click chemistry, a successful method for constructing novel pharmacophores through a series of dependable chemical reactions, has played a significant role in the discovery and optimization of drug leads (Kolb et al. 2001). In 2006, researchers have (Marik and Sutcliffe 2006) pioneered the introduction of Cu-catalyzed azide alkyne cycloaddition (CuAAC) reaction into the synthesis and labeling of radiopharmaceuticals, thereby introducing a novel approach for radionuclide labeling. As the development of radiopharmaceuticals progressed, an increasing number of radionuclides have been employed for click chemistry labeling (Zhong et al. 2023), including both radiodiagnostic radionuclides such as ^99m^Tc, ^18^F, and ^68^Ga and radiotherapeutic radionuclides such as ^188^Re and ^177^Lu (Colombo and Bianchi 2010; Mindt et al. 2008; Choy et al. 2017; Evans et al. 2014; Quigley et al. 2022; Wang et al. 2012). In addition, the synthesis of radiotracers targeting PSMA receptors for use as PRLT by click chemistry has been widely used, e.g., Verena i Böhmer synthesised and in vivo evaluated F-18 labeled PSMA-targeted “^18^F-PSMA-MIC” radiotracers by copper(I)-catalyzed azide-alkane cycloaddition and demonstrated that the binding affinity could be improved by copper(I)-catalyzed azide-alkane cycloaddition to promote alkane binding in PSMA (Böhmer et al. 2020); James Kelly developed high-affinity PSMA inhibitors labeled with F-18 by click chemistry, and showed that the radiosynthesis of ^18^F-labeled triazoles is simple and highly productive, with high PSMA affinity and specific uptake (Kelly et al. 2017).

In this study, the potential of click chemistry in simplifying and unifying the synthesis of metal radiopharmaceuticals was proposed and explored using an ^177^Lu-labeled PSMA-targeting molecular probe. The ^177^Lu-labeled PSMA ligand was achieved under mild conditions by incorporating a triazole ring into the molecular structures of DOTA and PSMA using click chemistry, named ^177^Lu-DOTA-CC-PSMA. The effect of this triazole ring on the overall drug properties of the complex was preliminarily investigated, and its biological properties were studied in mice with PSMA-positive tumors.

## Materials and methods

### Main instruments

The main equipment used in this study included a 1260 Infinity II high-performance liquid chromatography (HPLC) (Agilent Technologies, Inc., USA), an AR2000 thin-layer chromatography (TLC) scanner (Eckert & Ziegler Radiopharma, Inc., USA), a Flow-RAM radioactivity detector (LabLogic Systems Ltd., UK), a Wizard 2470 gamma counter (Perkin-Elmer Instruments Inc., USA), and a X-cube and γ-cube integrated SPECT/CT scanner (Molecubes, Belgium).

### Radiosynthesis of ^177^Lu-DOTA-CC-PSMA and ^177^Lu-PSMA-617

**Scheme 1:**
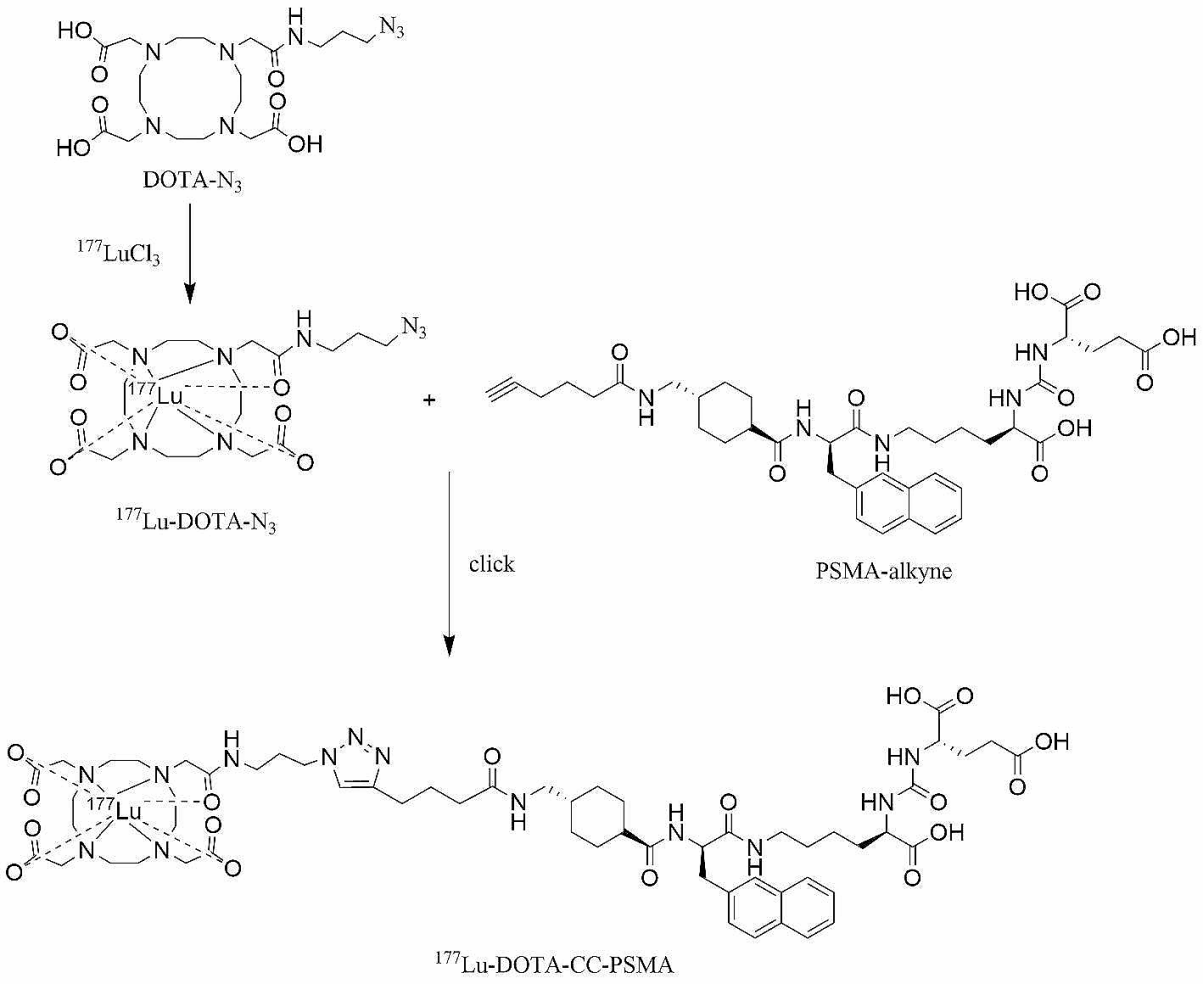
Radiosynthesis of ^177^Lu-DOTA-CC-PSMA

Detailed methods and results on the cold labeling of ^175^Lu-DOTA-CC-PSMA are presented in the Supplementary Data (Figs. S1 and S2). The radiosynthesis scheme of ^177^Lu-DOTA-CC-PSMA is shown in Scheme 1. ^177^LuCl_3_ was obtained from Isotopia Molecular Imaging Ltd (Petach Tikva, Israel). Briefly, DOTA-N_3_ (0.41 mmol/L, 150 µL, 62 nmol) was added to ^177^LuCl_3_ (25 µL, 0.67 GBq) in a sealed vial. The reaction was allowed to proceed at 95 °C for 15 min. The labeling rate was evaluated by HPLC method [A: H_2_O-0.1% trifluoroacetic acid (TFA), B: CH_3_CN-0.1% TFA, 1-1-10-10-1-1% B (0-5-10-14-15–17 min), 1 mL/min]. A solution of PSMA-alkyne (0.93 mmol/L, 95 µL, 89 nmol), CuSO_4_ (20.00 mmol/L, 43 µL, 860 nmol), and sodium ascorbate (94.40 mmol/L, 45 µL, 4250 nmol) was added to the freshly prepared ^177^Lu-DOTA-N_3_ mixture with final concentration. The resulting mixture was heated at 37 °C for 60 min. The tracer mixture was passed through a Sep-Pak cartridge (Waters, Sep-Pak C18 column) and rinsed with sterile water for injection to remove impurities and salts, and the tracer was eluted with ethanol. Finally, the solution was diluted with 0.5 M HAc-NaAc buffer solution (pH 5.2) and filtered through a 0.22-µm disposable sterile filter to obtain an injectable solution [< 8% (v: v) ethyl alcohol (EtOH)]. The radiochemical purity (RCP) was evaluated by the HPLC method [A: H_2_O-0.1% TFA, B: CH_3_CN-0.1% TFA, 20-40-40-100-20-20% B (0-20-22-27-30–35 min), 1 mL/min].

^177^Lu-PSMA-617 was prepared by successively adding ^177^LuCl_3_ solution (5 µL, 40.7 MBq), PSMA-617 (0.22 mmol/L, 30 µL, 6.7 nmol; prepared in 0.5 M HAc-NaAc buffer, pH 5.2), and gentisic acid (65.00 mmol/L, 40 µL, 2600 nmol) in a reaction flask. The mixture was mixed evenly and incubated for 15 min at 95 °C. Finally, the solution was cooled and diluted with 930 µL of sodium ascorbate (0.28 M) solution and filtered through a 0.22-µm disposable sterile filter to obtain an injectable solution. The RCP was evaluated by the HPLC method [A: H_2_O-0.1% TFA, B: CH_3_CN-0.1% TFA, 20-40-40-100-20-20% B (0-20-22-27-30–35 min), 1 mL/min].

### Stability test

To evaluate the stability of ^177^Lu-DOTA-CC-PSMA, samples were mixed with HAc-NaAc buffer solution or serum and incubated at 25–37 °C. Samples were taken at 0, 1, 4, 24, 48, 72, 120 and 144 h (*n* = 3) and analysed with radio-HPLC [A: H_2_O-0.1% TFA, B: CH_3_CN-0.1% TFA, 20-40-40-100-20-20% B (0-20-22-27-30–35 min), 1 mL/min] or radio-TLC [iTLC-SA (Agilent), 0.9% NaCl/HCl, 100:0.5] and further plotted in OriginPro 2019b.

### Determination of the partition coefficient

The partition coefficient was determined by mixing the labeled compound ^177^Lu-DOTA-CC-PSMA or ^177^Lu-PSMA-617 (10 µL) with n-octanol (600 µL) and phosphate buffer (590 µL, 0.1 M, pH 7.4) in an Eppendorf microcentrifuge tube. The mixture was vigorously stirred for 2 min at room temperature and was centrifuged at 8,000 rpm for 3 min. Samples in triplicate from n-octanol and aqueous layer were obtained, and were counted by γ-counter (*n* = 6). The partition coefficients were calculated using the following equation: logD_7.4_ = log (activity concentration in n-octanol)/(activity concentration in aqueous layer).

### Pharmacokinetics

^177^Lu-DOTA-CC-PSMA (3.7 MBq in 100 µL) or ^177^Lu-PSMA-617 (3.7 MBq in 100 µL) was administered intravenously via the tail into BALB/c male mice (*n* = 6), and a venous blood sample (5 µL) was collected from the tail end of each mouse at different time points after injection (2, 4, 6, 10, 15, 30, 60, 120, and 240 min). The radioactivity of the blood samples was measured by γ-counter, and the pharmacokinetics data were obtained using the DAS 2.0 software.

### Tumor model

All experiments were approved by the Committee on the Management and Use of Laboratory Animals of Shanghai Vista Pharmaceutical Technology Co. Ltd. (Institutional Animal Care and Use Committee number Vista-IA-2-1-2306-01), and all methods were carried out in accordance with relevant guidelines and regulations. The reporting in this manuscript adhered to the recommendations in the ARRIVE guidelines.

For in vivo studies, 6-week-old athymic nu/nu CDX (22Rv1) model male mice (subcutaneously inoculating 1 × 10^6^ 22Rv1 cells at the base of the right forelimb) were obtained from Nanchang Royo Biotech Co. Ltd. The mice were kept in individually ventilated cages under standard conditions with food and water provided ad libitum. Housing conditions were as following: dark/light cycle 12/12 h, ambient temperature around 21–22 °C and humidity between 40 and 70%. Biodistribution and SPECT/CT imaging were performed 3–4 weeks after cell inoculation (when the tumor reached a size of approximately 400 mm^3^). ^99m^Tc-HYNIC-PSMA SPECT/CT imaging was performed to verify PSMA-avidity of CDX models before the start of the experiment. Details on SPECT/CT acquisition process and PSMA-avidity assessment are provided in Supplementary Data (Fig. S3).

### In vivo biodistribution studies

The 22Rv1 tumor-bearing mice were injected in the lateral tail vein with ^177^Lu-DOTA-CC-PSMA (*n* = 4/group, 3.7 MBq in 100 µL per mouse) or ^177^Lu-PSMA-617 (*n* = 4/group, 3.7 MBq in 100 µL per mouse) under isoflurane anesthesia (1-2% isoflurane) and sacrificed by cervical dislocation 0.5, 1, 4, and 24 h later. The mice were kept under persistent anesthesia and warm throughout the injection period. Blood (^177^Lu-DOTA-CC-PSMA), organs (^177^Lu-DOTA-CC-PSMA), and tumors (^177^Lu-DOTA-CC-PSMA and ^177^Lu-PSMA-617) were collected and weighed. For the tumor specimens, liquid necrotic areas were discarded, and only solid tumor parts were examined. Activity content was assessed by γ-counter. Tissue counts and injected dose for individual mice were decay-corrected to the time of euthanasia. Tissue uptake was expressed as the percentage injected dose per gram of tissue (% injected dose/gram tissue, %ID/g). Values of tumor uptake were divided by muscle (blood, kidneys) uptake for each animal to calculate the tumor-to-muscle (blood, kidneys) [T/M (B, K)] ratio. The biodistribution data and target/non-target (T/NT) ratios were reported as mean values with the standard deviation (SD).

### SPECT/CT imaging

A total 4 mice with subcutaneous 22Rv1 tumors were scanned as exemplary cases for in vivo SPECT/CT imaging. Under isoflurane anesthesia (1-2% isoflurane) ^177^Lu-DOTA-CC-PSMA (18.5 MBq in 100 µL per mouse) injection and whole-body imaging was performed 0.5, 1, 2, 4, 6 and 24 h after injection with modular SPECT/CT scanner. The mice were placed in the supine position on the scanning bed post-injection, respectively, and were scanned using modular high-resolution CT (image acquisition parameters: CT, tube voltage: 50 kV; tube current: 30 µA), which was anaesthetised for 5 min using 1% isoflurane. After the CT acquisition, the mice were placed supine on the scanning bed and scanned using modular high-resolution SPECT (image acquisition parameters: SPECT, algorithm: maximum likelihood algorithm; peak: 140 keV; isometric voxel size: 500 μm), and anaesthesia was applied with 1% isoflurane for 28 min. Images were constructed using maximum likelihood algorithm (50 iterations) and the data were corrected for attenuation. Image processing and analysis were performed using VivoQuant software (Invicro) to form hybrid SPECT/CT images in transverse, coronal and sagittal planes.

### Data analysis and statistics

All statistical analyses were performed using SPSS 23.0 software (SPSS Inc.), and graphs were plotted using OriginPro 2019b (OriginLab Inc.). Quantitative data are expressed as the mean ± SD. Independent samples of tumor uptake for each organ, as well as for tumor tissue and T/M (T/B, T/K) ratio, tracer uptakes at each time point were compared with Kruskal Wallis H test, and Mann-Whitney-U test was used as post-hoc test in pairwise comparisons of variables with significant results. Two-sided *p*-values of < 0.05 were considered statistically significant.

## Results

### Radiochemistry

**Fig. 1:**
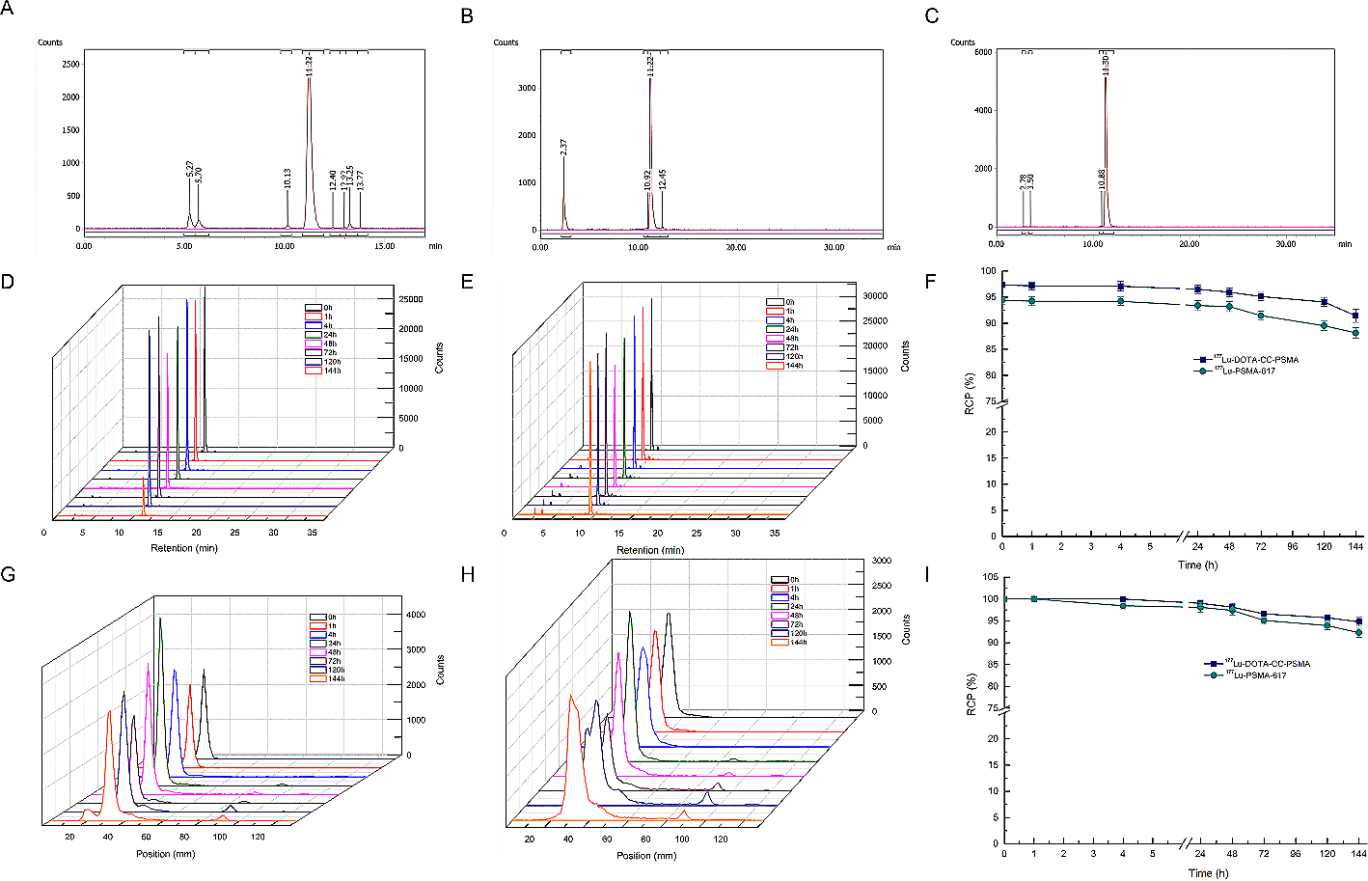
Radio-HPLC profile of (**A**) ^177^Lu-DOTA-N_3_, (**B**) ^177^Lu-DOTA-CC-PSMA, and (**C**) purified ^177^Lu-DOTA-CC-PSMA. Multi-temporal superimposed Radio-HPLC profile of (**D**) ^177^Lu-DOTA-CC-PSMA, (**E**) ^177^Lu-PSMA-617, and (**F**) RCP of ^177^Lu-DOTA-CC-PSMA and ^177^Lu-PSMA-617 measured within 144 h in HAc-NaAc buffer. Multi-temporal superimposed Radio-TLC profile of (**G**) ^177^Lu-DOTA-CC-PSMA, (**H**) ^177^Lu-PSMA-617, and (**I**) RCP of ^177^Lu-DOTA-CC-PSMA and ^177^Lu-PSMA-617 measured within 144 h in serum

Radiolabeling of the precursor was accomplished by first reacting ^177^LuCl_3_ with DOTA-N_3_, followed by a click-chemistry step to assemble the intact ^177^Lu-DOTA-CC-PSMA (Scheme 1). ^177^LuCl_3_ and DOTA-N_3_ were incubated in a sodium acetate buffer (pH 5.2) at 95 °C for 15 min. DOTA-N_3_ incorporated ^177^LuCl_3_ readily with a labeling rate of 86.56% ± 1.26% (*n* = 6), as determined by radio-HPLC (Fig. 1A), and the CUAAC was initiated by adding PSMA-alkyne into the ^177^Lu-DOTA-N_3_ mixture at 37 °C for 60 min. The yield of ^177^Lu-DOTA-CC-PSMA was 77.07% ± 0.03% (*n* = 6), as determined by radio-HPLC (Fig. 1B); the RCP was 97.62% ± 1.49% (*n* = 6) after the purification according to radio-HPLC analysis (Fig. 1C); and the molar activity was 5.0 MBq/nmol. As shown in Table 1, the reaction temperature and the RCP of ^177^Lu-DOTA-CC-PSMA were superior to those of ^177^Lu-PSMA-617. The stabilities of ^177^Lu-PSMA-617 and ^177^Lu-DOTA-CC-PSMA in HAc-NaAc buffer (25 °C) and serum (37 °C) were investigated, and the results showed that the RCP (> 95%) of ^177^Lu-DOTA-CC-PSMA was superior to that of ^177^Lu-PSMA-617 over an observation period of 144 h (Fig. 1D-I), which indicates that ^177^Lu-DOTA-CC-PSMA is of high stability.

The logD_7.4_ values were − 3.99 ± 0.32 for ^177^Lu-DOTA-CC-PSMA and − 4.21 ± 0.08 for ^177^Lu-PSMA-617, suggesting that ^177^Lu-DOTA-CC-PSMA was highly hydrophilic and could be rapidly eliminated from blood.

**Table 1:** Radiochemistry of ^177^Lu-PSMA-617 and ^177^Lu-DOTA-CC-PSMA

Radiopharmaceutical	Temp	pH	Time	Labeling rate	RCP
^177^Lu-PSMA-617	95 ℃	5.0	15 min	94.29 ± 0.01%	94.29 ± 0.01%
^177^Lu-DOTA-N_3_	95 ℃	5.2	15 min	86.56 ± 1.26%	-^#^
^177^Lu-DOTA-CC-PSMA	37 ℃	5.2	15 min	34.49 ± 0.06%	-
			30 min	52.16 ± 0.03%	-
			60 min	77.07 ± 0.04%	97.62 ± 1.49%

^#^Unpurified, used directly in the next step

### Biodistribution and tumor uptake

**Fig. 2:**
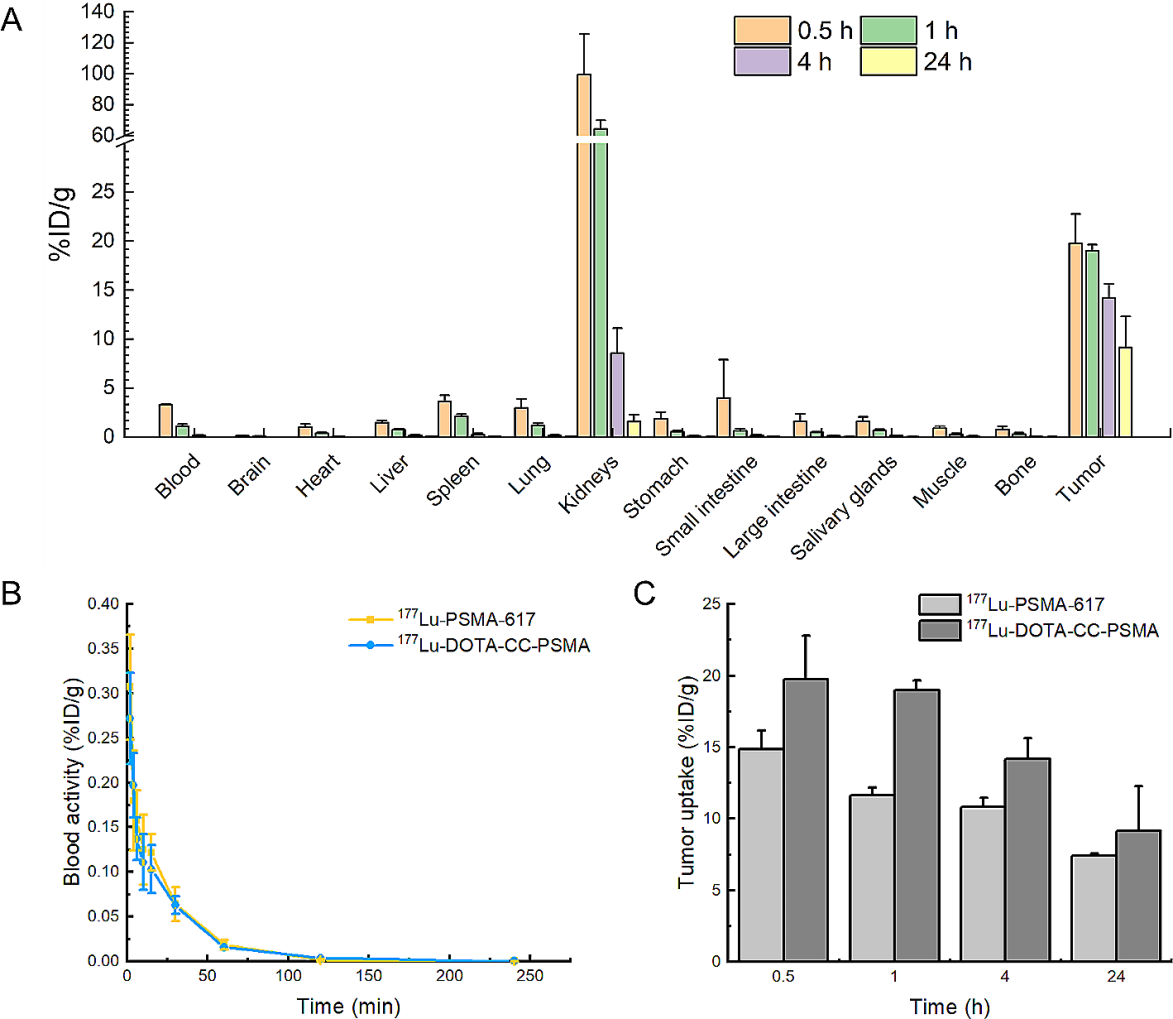
(**A**) Biodistribution of ^177^Lu-DOTA-CC-PSMA. Data were obtained 0.5, 1, 4, and 24 h after ^177^Lu-DOTA-CC-PSMA injection. Values are expressed as %ID/g for organs and tissues. Values are shown as mean ± SD; *n* = 4. (**B**) Blood drug concentration-time curves for ^177^Lu-PSMA-617 and ^177^Lu-DOTA-CC-PSMA. Values are shown as mean ± SD; *n* = 6. (**C**) Tumor uptake of ^177^Lu-PSMA-617 and ^177^Lu-DOTA-CC-PSMA over time. Values are shown as mean ± SD; *n* = 4

In vivo biodistribution data showed highly specific tumor uptake of ^177^Lu-DOTA-CC-PSMA in PSMA-positive tumors (Table 2; Fig. 2A).

Tumor uptake of ^177^Lu-DOTA-CC-PSMA peaked at 30 min (19.75 ± 3.02%ID/g). However, ^177^Lu-DOTA-CC-PSMA showed the highest tumor-to-muscle (blood) ratio at 24 h (T/M: 801.21 ± 299.73; T/B: 296.30 ± 71.82), compared with the time point of the tumor uptake peak (T/M: 22.70 ± 6.07; T/B: 6.29 ± 1.46), indicating fast initial uptake followed by slow tumor activity washout paralleled by a stronger washout in the muscle (blood) (Table 2). Still, the T/M (B) ratio was greater than 1 at all of the examined time points (Table 1). ^177^Lu-DOTA-CC-PSMA was excreted via the renal route. Among normal organs, the kidneys showed the highest uptake (1.58 ± 0.70%ID/g at 24 h after injection), while others showed very low radioactivity accumulation and rapid elimination (Fig. 2A).

The blood drug concentration-time curves of the probe ^177^Lu-PSMA-617 and ^177^Lu-DOTA-CC-PSMA in healthy mice are shown in Fig. 2B. The curves showed that the pharmacokinetics of ^177^Lu-DOTA-CC-PSMA and ^177^Lu-PSMA-617 were essentially the same (*P* > 0.05), with rapid clearance from the blood and rapid distribution to all tissues and organs in the body. In addition, we calculated the blood clearance half-life of the radiopharmaceuticals synthesised by the two methods, which was shorter at 15.16 min for ^177^Lu-DOTA-CC-PSMA compared to ^177^Lu-PSMA-617 (17.18 min).

In addition, we compared the tumor uptake of ^177^Lu-DOTA-CC-PSMA (click chemistry method) and that of ^177^Lu-PSMA-617 (conventional labeling method) and found that the tumors had significantly higher uptake of the click chemistry-synthesized ^177^Lu-DOTA-CC-PSMA (Fig. 2C, *P* < 0.05 within 24 h).

**Table 2:** In vivo evaluation of tumor uptake (^177^Lu-DOTA-CC-PSMA and ^177^Lu-PSMA-617) and biodistribution of ^177^Lu-DOTA-CC-PSMA in mice model. (ID%/g, mean ± SD)

Organ	0.5 h (%ID/g)	1 h (%ID/g)	4 h (%ID/g)	24 h (%ID/g)	*p*-value
Blood	3.27 ± 0.09	1.08 ± 0.25	0.16 ± 0.04	0.03 ± 0.00	0.002
Brain	0.14 ± 0.01	0.08 ± 0.03	0.02 ± 0.00	0.01 ± 0.00	0.002
Heart	1.03 ± 0.32	0.39 ± 0.08	0.06 ± 0.01	0.02 ± 0.00	0.002
Liver	1.46 ± 0.25	0.74 ± 0.07	0.18 ± 0.06	0.07 ± 0.02	0.003
Spleen	3.61 ± 0.66	2.11 ± 0.22	0.24 ± 0.14	0.05 ± 0.02	0.003
Lung	2.98 ± 0.90	1.16 ± 0.28	0.18 ± 0.04	0.06 ± 0.01	0.003
Kidneys	99.49 ± 26.18	64.50 ± 5.38	8.56 ± 2.55	1.58 ± 0.70	0.003
Stomach	1.86 ± 0.70	0.55 ± 0.11	0.12 ± 0.03	0.03 ± 0.02	0.003
Small intestine	4.00 ± 3.90	0.63 ± 0.23	0.17 ± 0.06	0.05 ± 0.01	0.003
Large intestine	1.62 ± 0.75	0.47 ± 0.07	0.14 ± 0.01	0.04 ± 0.01	0.003
Salivary glands	1.62 ± 0.44	0.65 ± 0.15	0.15 ± 0.03	0.04 ± 0.01	0.003
Muscle	0.91 ± 0.22	0.28 ± 0.10	0.08 ± 0.05	0.01 ± 0.00	0.002
Bone	0.76 ± 0.34	0.30 ± 0.15	0.07 ± 0.02	0.03 ± 0.02	0.004
Tumor	19.75 ± 3.02	19.00 ± 0.65	14.20 ± 1.45	9.14 ± 3.16	0.006
Tumor/Blood^#^	6.29 ± 1.46	18.20 ± 3.35	95.69 ± 20.19	296.30 ± 71.82	0.003
Tumor/Muscle^#^	22.70 ± 6.07	73.06 ± 22.71	213.33 ± 79.99	801.21 ± 299.73	0.003
Tumor/Kidneys^#^	0.21 ± 0.09	0.30 ± 0.02	1.74 ± 0.36	6.34 ± 2.19	0.004

*n* = 4, if not indicated otherwise

^#^*n* = 4, and ratio instead of ID%/g. *p*-values are given for comparison of each time point for each tissue. Only mice with tumor inoculation have been used in this analysis

### SPECT/CT imaging

**Fig. 3:**
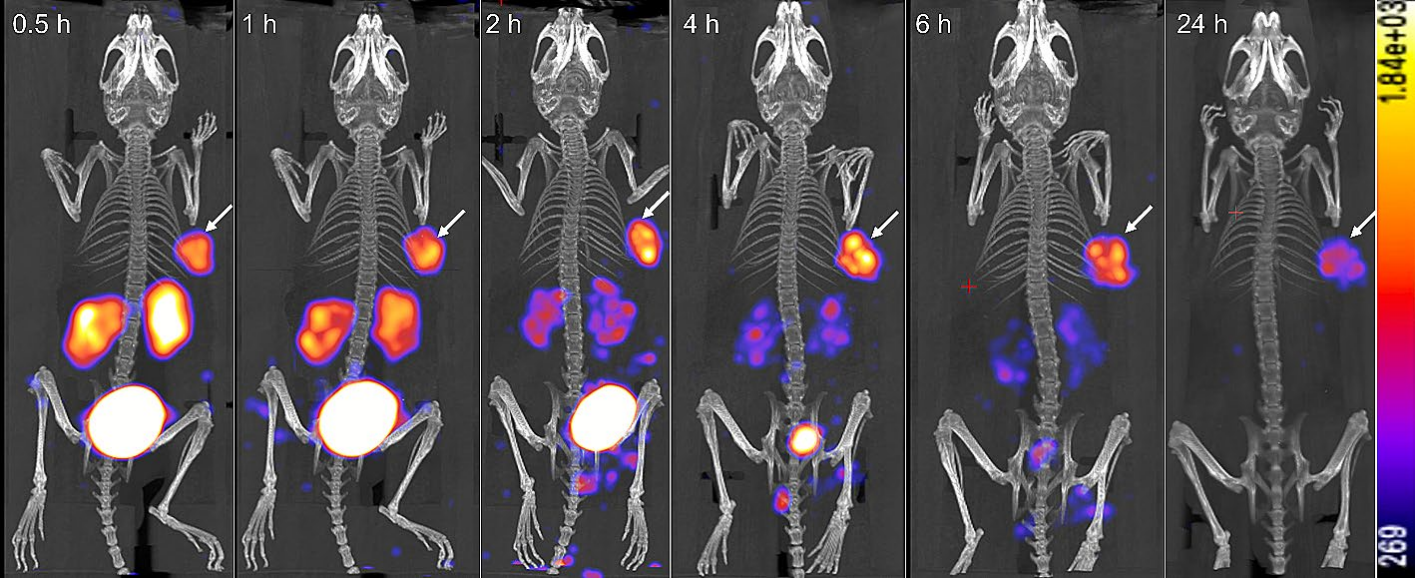
In vivo ^177^Lu-DOTA-CC-PSMA of a PSMA-positive tumor model. Whole-body scans at 0.5, 1, 2, 4, 6, and 24 h after injection of ^177^Lu-DOTA-CC-PSMA (22Rv1 tumor-bearing mouse) are shown, with clearly visible renal accumulation, low uptake in other normal organs, and tumor uptake peaking at 2 h and remaining at 24 h. The tumor is marked with a white arrow

SPECT/CT images (Fig. 3) showed high tumor uptake in 22Rv1 tumor-bearing mice at all time points, peaking at 2 h, and tumor uptake even after 24 h, indicating that ^177^Lu-DOTA-CC-PSMA has a high tumor affinity with good accumulation and retention capacity. The uptake of ^177^Lu-DOTA-CC-PSMA was moderate in the kidneys and low in other normal organs, with a gradual decrease in uptake over time, and the radioactivity essentially disappeared from the kidneys and bladder after 24 h, whereas ^177^Lu-DOTA-CC-PSMA was further enriched and retained in the PSMA-positive tumors. High T/M ratios and high T/K ratios indicated good non-target organ clearance efficiency and high T/NT ratios. Thus, targeting molecular probes synthesized by click chemistry with good tumor uptake and rapid clearance shows significant potential in simplifying and unifying the synthesis of metal radiopharmaceuticals.

## Discussion

This study explored the potential of click chemistry in simplifying and unifying the synthesis of metal radiopharmaceuticals using ^177^Lu-labeled PSMA-targeting molecule probe as an example. ^177^Lu has a long half-life and can release γ-rays, and these γ-rays in SPECT imaging and external dose assessment have the potential for early diagnosis of tumors, recurrence, and metastatic foci (Liu and Chen 2019). As the pairing metal nuclide of ^177^Lu, the positronic nuclide ^68^Ga has a similar labeling chemistry and can often be labeled with the same drug precursor, and thus it can be prepared for diagnosis and treatment integration of radiopharmaceuticals (Chen et al. 2018). In a similar way, the proposed protocol is suited for ^68^Ga-labeling of bioactive molecules. This study achieves a significant reduction in time compared to other click chemistry methods (Choy et al. 2017), which is not significant for Lu-177 labeling, but as a methodological study is significant for the improvement of the radiochemical yield of other short half-life nuclides, e.g. Ga-68.

The synthesis of ^177^Lu-DOTA-CC-PSMA is straightforward and expands the scope of PSMA-617 as a labeling precursor, which simplifies the labeling process by avoiding high-temperature reaction conditions compared with conventional ^177^Lu labeling. In this study, the click chemistry method was employed to incorporate a triazole ring into both the DOTA and PSMA molecular structure. The effect of this triazole ring on the overall drug properties of the complex was subsequently investigated, and its biological properties were studied in mice with PSMA-positive tumors. The results showed that ^177^Lu-DOTA-N_3_ was successfully labeled with PSMA-alkyne compounds after using click chemistry, and the labeling process was simple and efficient, with mild conditions and high RCP. The ^177^Lu-DOTA-CC-PSMA in this study showed good in vitro and in vivo stability, which was still maintained at more than 90% within 144 h, which is more consistent with the stability of ^177^Lu-PSMA-617 (> 90% within 144 h). ^177^Lu-DOTA-CC-PSMA could also maintain stability in vivo, indicating that it can be used for in vivo studies.

^177^Lu-DOTA-CC-PSMA is a PSMA-targeted nuclear drug with translational therapeutic promise for PRLT. ^177^Lu-DOTA-CC-PSMA is of good hydrophilic and low lipid-soluble, so it does not easily cross the blood-brain barrier, and the brain tissue is exposed to less radiation. Our pharmacokinetic results showed that ^177^Lu-DOTA-CC-PSMA could be cleared from the blood and rapidly distributed to all tissues and organs in the body, providing a lower blood background signal during imaging, which was conducive to the formation of a high T/B ratio and clear SPECT images. The biodistribution and SPECT/CT imaging results in the 22Rv1 tumor-bearing mice, especially the significant renal excretion, suggested that ^177^Lu-DOTA-CC-PSMA was efficiently cleared from the blood and other organs, which is an important aspect of in vivo imaging with targeted molecular probes. Meanwhile, the results of the biodistribution and SPECT/CT imaging showed that ^177^Lu-DOTA-CC-PSMA exhibited superior uptake and retention within the tumor, good non-target organ clearance efficiency, and high T/NT ratio. ^177^Lu-DOTA-CC-PSMA is of the advantage of achieving high accumulation within the tumour, with potential therapeutic value.

In conclusion, the labeling of ^177^Lu-DOTA-CC-PSMA synthesized by click chemistry is simple and easy, with mild labeling conditions, qualified quality control, and high labeling rate; ^177^Lu-DOTA-CC-PSMA has ideal biodistribution, most of which is excreted via the kidneys; and it offers rapid blood clearance and good stability, demonstrating the potential of click chemistry to unify the synthesis of metal radiopharmaceuticals. However, only the popular ^177^Lu-labeled PSMA-targeting molecular probe was used as an example in this study, and more studies on metal nuclide-labeled targeting molecules were not carried out. The potential of click chemistry to unify the synthesis of metal radiopharmaceuticals has yet to be verified for more metal nuclides through the click-chemistry-labeled targeting molecular probes.

## Conclusion

In this study, we proposed a scheme to avoid the loss of targeting properties during the degeneration and inactivation of targeting molecules in the process of producing metal nuclide-conjugated radiopharmaceuticals and validated it with ^177^Lu-labeled PSMA-targeting molecules. A novel ^177^Lu-labeled PSMA-targeted molecular probe, designated as ^177^Lu-DOTA-CC-PSMA, was successfully synthesized through the click chemistry of ^177^Lu-DOTA-N_3_ with PSMA-alkyne compound. The stability and tumor uptake of ^177^Lu-DOTA-CC-PSMA synthesized by click chemistry were higher and with consistent pharmacokinetics, compared with ^177^Lu-PSMA-617 synthesized by conventional methods. Consequently, metal nuclide-coupled radiopharmaceuticals, represented by ^177^Lu-DOTA-CC-PSMA, demonstrate the potential of click chemistry to unify the synthesis of metal radiopharmaceuticals.

### Electronic supplementary material

Below is the link to the electronic supplementary material.

Supplementary Material 1

## Data Availability

The authors declare that the data supporting the findings of this study are available within the paper and its Supplementary Information files. Should any raw data files be needed in another format they are available from the corresponding author upon reasonable request.

## Abbreviations

PSMA: Prostate-specific membrane antigen
PRLT: PSMA-targeted radioligand therapy
SPECT: Single-photon emission computed tomography
CT: Computed tomography
HPLC: High performance liquid chromatography
TLC: Thin-layer chromatography
ROI: Region of interest
T/M: Tumor to muscle
T/K: Tumor to kidney
T/B: Tumor to blood
T/NT: Target/non-target
RCP: Radiochemical purity
%ID/g: % injected dose/gram tissue
CuAAC: Cu-catalyzed azide alkyne cycloaddition
TFA: Trifluoroacetic acid
EtOH: Ethyl alcohol
