# Supplementary material for: The development of 177Lu-DOTA-CC-PSMA following a unified “Click Chemistry” protocol of synthesizing metal nuclide-conjugated radiopharmaceuticals
Source: EJNMMI Radiopharm Chem. 2024 Jul 31;9:56. doi: 10.1186/s41181-024-00287-7 (PMC11291776; doi:10.1186/s41181-024-00287-7)
Supplement: Supplementary file 1 — Supplementary Material 1 [file 41181_2024_287_MOESM1_ESM.docx]

Supplementary Information

**The development of ^177^Lu-DOTA-CC-PSMA following a unified "Click Chemistry" protocol of synthesizing metal nuclide-conjugated radiopharmaceuticals**

Xiaobei Zheng^1,2,3#^, Shuai Xue^1,4#^, Zhongqi Zhao^1,2^, Shuxin Jin^3^, Shuhua He^1,2,3^, Lina Jia^1,3^, Zheng Li^1,3^, Christian Vanhove^5^, Filip De Vos^6^, Zijun Kuang^3^, Tiantian Wang^3^, Sara Neyt^5*^, Lan Zhang^1*^, Xiao Li^1,4*^

1. Shanghai Institute of Applied Physics, Chinese Academy of Sciences, Shanghai 201800, China
2. University of Chinese Academy of Sciences, Beijing 100049, China
3. Shanghai Vista Pharmaceutical Technology Co. Ltd，Shanghai 201800, China
4. Department of Nuclear Medicine, Pudong Hospital, Fudan University, Shanghai 201399, China
5. Institute of Biomedical Engineering and Technology, Faculty of Engineering and Architecture, Ghent University, Ghent 9000, Belgium
6. Department of Radiopharmacy, Faculty of Pharmacy, Ghent University, Ghent 9000, Belgium

^#^These authors contributed equally to this work

^*^Correspondence to: Sara Neyt, Institute Biomedical Engineering and Technology, Faculty of Engineering and Architecture, Ghent University, Ghent 9000, Belgium; Lan Zhang, Shanghai Institute of Applied Physics, Chinese Academy of Sciences, Shanghai 201800, China; Xiao Li, Shanghai Institute of Applied Physics, Chinese Academy of Sciences, Shanghai 201800, China.

E-mail addresses: Sara.Neyt@UGent.be (Sara Neyt); zhanglan@sinap.ac.cn (Lan Zhang); lixiao@sinap.ac.cn (Xiao Li).

**Synthesis of ^175^Lu-DOTA-N_3_**

In a reaction flask, the mixture of ^175^LuCl_3_ (50 μL, 0.07 M) and DOTA-N_3_ (500 μL, 0.004 M) is conducted. The reaction mixture was 95 ℃ for 30 min. The residue was purified by HPLC (t_R_ = 10.57 min), Electrospray ionization-mass spectrometry: m/z = 659.45 [M+H]^+^ (C_19_H_31_LuN_8_O_7_, calculated molecular weight [MW] = 658.17).


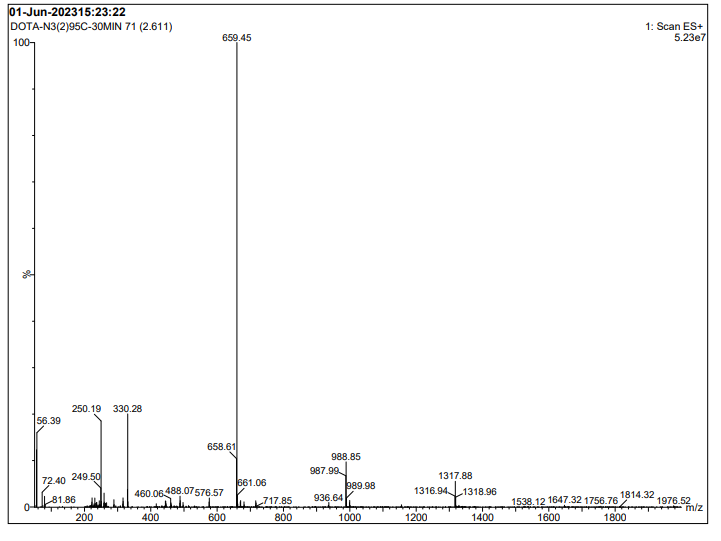


**Figs.S1** Liquid chromatograph-mass spectrometer profile of ^175^Lu-DOTA-N_3_

**Synthesis of ^175^Lu-DOTA-CC-PSMA**

The solution of PSMA-alkyne (50 μL, 47 nmol), ^175^Lu-DOTA-N_3_ (8.3 μL，31.3 nmol), CuSO_4_ (6.8 μL, 125.2 nmol), and Sodium ascorbate (10.2 μL, 1878 nmol) was mixed at 60 ℃ for 60 min. The residue was purified by HPLC (t_R_ = 28.10 min), Electrospray ionization-mass spectrometry: m/z = 705.16 [M+2H]^2+^/2 (C_58_H_82_LuN_13_O_17_, calculated molecular weight [MW] = 1407.54).


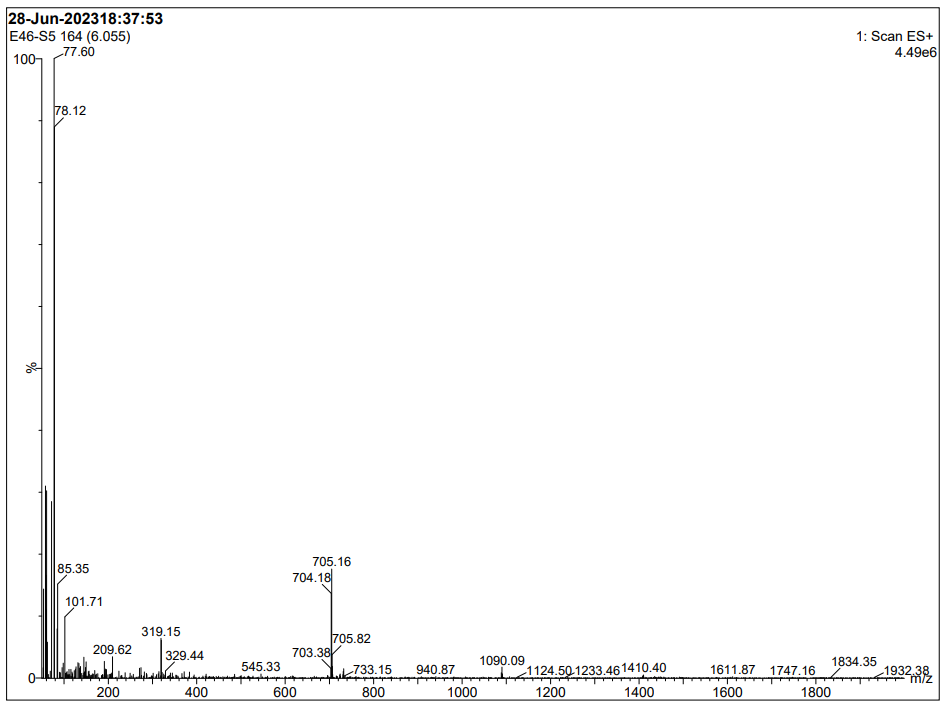


**Figs.S2** Liquid chromatograph-mass spectrometer profile of ^175^Lu-DOTA-CC-PSMA

**Radiosynthesis of ^99m^Tc-HYNIC-PSMA**

Briefly described as follows, 25 mCi Na^99m^TcO_4_ were added to HYNIC-PSMA kit (Shanghai Nice-Labeling Biotechnology Co, Ltd), and the reaction was carried out at 100 °C for 10 min. The labeling rate was greater than 99% without purification.

**SPECT/CT imaging**

Under isoflurane anesthesia (1%-2% isoflurane) ^99m^Tc-HYNIC-PSMA (22.9 MBq in 100 μL per mouse) injection and whole-body imaging was performed 1.5 h after injection with modular SPECT/CT scanner (Molecubes, Belgium). The mice were placed in the supine position on the scanning bed post-injection, respectively, and were scanned using modular high-resolution CT (image acquisition parameters: CT, tube voltage: 50 kV; tube current: 30 µA), which was anaesthetised for 5 min using 1% isoflurane. After the CT acquisition, the mice were placed supine on the scanning bed and scanned using modular high-resolution SPECT (image acquisition parameters: SPECT, algorithm: maximum likelihood algorithm; peak: 140 keV; isometric voxel size: 500 µm), and anaesthesia was applied with 1% isoflurane for 28 min. Images were constructed using maximum likelihood algorithm (50 iterations) and the data were corrected for attenuation. Image processing and analysis were performed using VivoQuant software (Invicro) to form hybrid SPECT images in transverse, coronal and sagittal planes.


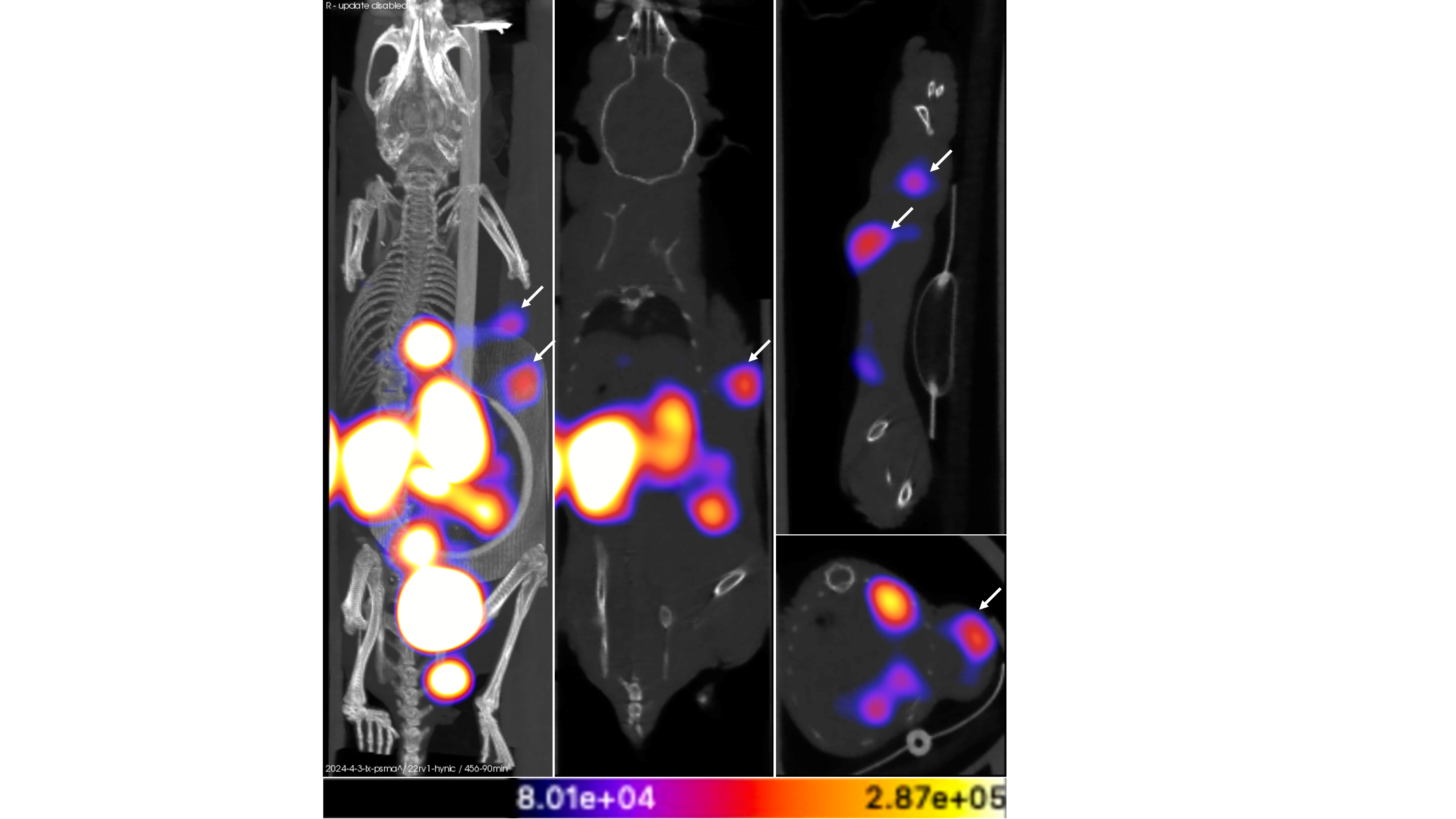


**Fig.S3** *In vivo* ^99m^Tc-HYNIC-PSMA of PSMA-positive (22RV1) tumor model. Tumor marked with a white arrow.
